# Live-Cell Optical Redox Imaging Reveals Metabolic Heterogeneity and Context-Dependent Responses to Metabolic Perturbation in TNBC Cells

**DOI:** 10.3390/metabo16090613

**Published:** 2026-08-27

**Authors:** He N. Xu, Jack Kollmar, Allison Podsednik, Mihiar Wannousse, Alexander Shestov, Roddy S. O’Connor, Joseph A. Baur, Julia Tchou, Rong Zhou, Lin Z. Li

**Affiliations:** 1 Department of Radiology, Perelman School of Medicine, University of Pennsylvania, Philadelphia, PA 19104, USA; podsednik@alumni.upenn.edu (A.P.); rongzhou@pennmedicine.upenn.edu (R.Z.); linli@pennmedicine.upenn.edu (L.Z.L.); 2 Department of Surgery, Perelman School of Medicine, University of Pennsylvania, Philadelphia, PA 19104, USA; jack.kollmar@pennmedicine.upenn.edu (J.K.); mihiar.wannousse@pennmedicine.upenn.edu (M.W.); julia.tchou@pennmedicine.upenn.edu (J.T.); 3 Center for Cellular Immunotherapies, Perelman School of Medicine, University of Pennsylvania, Philadelphia, PA 19104, USA; ashestov@pennmedicine.upenn.edu (A.S.); oconnorr@pennmedicine.upenn.edu (R.S.O.); 4 Department of Physiology, Institute for Diabetes, Obesity, and Metabolism, Perelman School of Medicine, University of Pennsylvania, Philadelphia, PA 19104, USA; baur@pennmedicine.upenn.edu

**Keywords:** NADH, FAD, ORR, ROS, mitochondrial redox state, wide-field metabolic imaging microscopy, OCR, ECAR, glutaminolysis, glycolysis

## Abstract

**Highlights:**

**What are the main findings?**

- Live-cell optical redox imaging (ORI) distinguished basal metabolic phenotypes and revealed distinct acute and temporal responses to glycolytic and glutaminolytic inhibition in TNBC cells.
- Integrating ORI with Seahorse metabolic flux analysis and imaging-based functional assays linked dynamic redox responses with pathway-specific metabolic and functional changes.

**What are the implications of the main findings?**

- ORI provides a practical, label-free approach for functional metabolic phenotyping at single-cell resolution.
- The multimodal ORI framework facilitates characterization of context-dependent metabolic responses and their functional consequences.

**Abstract:**

**Background/Objectives:** Triple-negative breast cancer (TNBC) exhibits substantial metabolic heterogeneity and plasticity, contributing to variable therapeutic responses. We investigated whether optical redox imaging (ORI) could characterize metabolic phenotypes, monitor responses to metabolic perturbation, and relate these responses to functional outcomes in TNBC cells. **Methods:** Four TNBC cell lines were treated with the lactate dehydrogenase A inhibitor FX11 or the glutaminase inhibitor CB-839, alone or in combination with paclitaxel. Intensity-based label-free ORI was performed and followed by imaging of fluorescent probes to assess mitochondrial membrane potential (MMP), re-active oxygen species (ROS), and cell number in the same dishes. Seahorse assays were used to evaluate mitochondrial respiration and glycolytic flux. **Results:** TNBC cell lines exhibited distinct basal redox phenotypes and differential responses to acute glycolytic and glutaminolytic perturbation. HCC1806 cells showed the strongest acute ORI responses to both FX11 and CB-839. Acute FX11 treatment induced a rapid reductive shift accompanied by ROS accumulation and loss of MMP, whereas CB-839 produced a more modest early reductive response without detectable ROS accumulation or MMP loss. Prolonged treatment revealed distinct temporal redox trajectories in HCC1806 cells: FX11-treated cells evolved from an acute reductive response toward a more oxidized state, while CB-839-treated cells transitioned from an early reductive shift to a sustained oxidized redox state accompanied by marked reductions in OCR and ECAR. Functionally, in two representative models (HCC1806 and MDA-MB-231), CB-839 reduced cell numbers and enhanced the anti-proliferative effect of paclitaxel, whereas FX11 had no significant effect on cell number despite inducing pronounced acute redox perturbations. **Conclusions:** Integrating ORI with metabolic flux assays and imaging-based functional measurements enables characterization of multiple dimensions of metabolic behavior, including basal phenotype, pathway-specific responsiveness, temporal redox responses, and treatment-associated outcomes. These findings support intensity-based wide-field ORI as a practical and accessible tool for probing metabolic heterogeneity and characterizing context-dependent metabolic responses in TNBC cells.

## 1. Introduction

Triple-negative breast cancer (TNBC), defined by the absence of estrogen receptor, progesterone receptor, and human epidermal growth factor receptor 2 (HER2) expression, is a highly heterogeneous and aggressive breast cancer subtype that lacks actionable molecular targets, making chemotherapy and more recently immunotherapy the primary systemic treatment [1,2,3]. Transcriptomic classification by Lehmann and colleagues delineated distinct TNBC subtypes based on gene expression clusters [4], underscoring biological diversity within this disease. However, emerging evidence suggests that metabolic phenotyping, including metabolic pathway-based stratification, may provide complementary and clinically predictive insight [5], as metabolic dependencies are functionally linked to therapeutic response.

Beyond static heterogeneity, metabolic plasticity—the ability of tumor cells to dynamically reprogram energy production and redox balance under therapeutic pressure—represents a key driver of drug resistance and treatment failure [6,7,8,9,10,11]. TNBC cells frequently exhibit elevated glycolysis to support rapid proliferation; however, they retain metabolic flexibility and can maintain or upregulate mitochondrial oxidative phosphorylation (OXPHOS) under conditions such as metastasis, cancer stemness, or metabolic stress, reflecting a context-dependent use of both glycolytic and mitochondrial pathways [9,12,13,14]. In addition to glycolysis, some TNBC cells are dependent on glutaminolysis, in which glutamine is converted to glutamate to fuel the tricarboxylic acid (TCA) cycle, particularly when glucose is limited, thereby supporting survival, growth, and metastatic reprogramming [10,15,16,17]. As such, combining glycolytic or glutaminolytic inhibitors with chemotherapy or immunotherapy may sensitize cancer cells to treatment, helping to overcome resistance and mitigate the impact of tumor heterogeneity [18,19,20,21]. Therefore, metabolic phenotyping approaches capable of capturing functional and dynamic metabolic states are critical for addressing variable treatment outcomes.

Live-cell optical redox imaging (ORI) leverages the intrinsic fluorescence of the metabolic coenzymes nicotinamide adenine dinucleotide (NADH) and flavin adenine dinucleotide (FAD) to quantify mitochondrial redox state in living cells. Because these coenzymes participate directly in OXPHOS, their fluorescence intensities and the derived mitochondrial redox ratio—optical redox ratio (ORR), defined as FAD/(NADH + FAD) or Fp/(NADH + Fp), where Fp denotes FAD-containing flavoproteins—provide a functional readout of cellular bioenergetics and metabolic state [22,23,24,25]. A higher ORR reflects a more oxidized mitochondrial redox state and is typically associated with greater reliance on oxidative metabolism, whereas a lower ORR indicates a more reduced redox state and a shift toward glycolytic metabolism. Unlike conventional bulk metabolomics, which provides a steady-state snapshot of metabolite abundance, ORI is a label-free technique that enables non-invasive, real-time assessment of metabolic dynamics at the single-cell level with compartmental resolution, allowing longitudinal analysis of metabolic heterogeneity and adaptive responses to therapeutic stress. ORI has been widely applied in multiple cancer models, including breast tumor cells, to characterize metabolic heterogeneity, therapeutic response, and aggressiveness [11,24,26,27,28,29,30,31,32,33,34,35]. These findings support the utility of ORI as a functional metabolic phenotyping platform in oncology studies.

Understanding how modulation of major metabolic pathways reshapes mitochondrial redox state and function in TNBC cells is critical for rational metabolic targeting. In particular, how metabolic inhibition interacts with standard chemotherapeutics such as paclitaxel (Taxol^®^) to influence mitochondrial redox balance and function, metabolic flux, and cell growth remains insufficiently characterized. Furthermore, whether quantitative metabolic imaging readouts can be directly linked to cell proliferation and function under metabolic stress has not been systematically explored. Addressing these gaps may reveal metabolic vulnerabilities and inform combination strategies that exploit mitochondrial redox stress in TNBC.

In this study, we investigated how inhibition of glycolysis and glutaminolysis influences mitochondrial redox state, metabolic flux, and cellular behavior in TNBC cells. Using live-cell ORI integrated with Seahorse metabolic flux analysis and imaging-based functional assays, we examined basal metabolic heterogeneity, acute and prolonged responses to pathway-specific metabolic perturbation, and the relationship between redox responses and treatment-associated functional outcomes. Through this approach, we sought to establish a functional framework for characterizing metabolic phenotypes and context-dependent responses to metabolic and chemotherapeutic stress in TNBC using ORI-derived redox metrics.

## 2. Materials and Methods

### 2.1. Cell Preparation and Treatment

Human TNBC cell lines MDA-MB-468, HCC1806, MDA-MB-436, and MDA-MB-231 were purchased from ATCC (Manassas, VA, USA). These cell lines were selected to represent biologically distinct TNBC phenotypes, including basal-like 1 (BL1; MDA-MB-468), basal-like 2 (BL2; HCC1806), and mesenchymal stem-like (MSL; MDA-MB-231 and MDA-MB-436) subtypes, thereby providing a panel for evaluating metabolic heterogeneity and differential responses to metabolic perturbation. MDA-MB-468 cells were routinely cultured in Leibovitz’s L-15 medium supplemented with 10% fetal bovine serum (FBS) (MilliporeSigma, St. Louis, MO, USA) at 37 °C in a CO_2_-free environment. All other TNBC cell lines were cultured in RPMI 1640 (Gibco, Cat# 11875085, Thermo Fisher Scientific, Waltham, MA, USA) supplemented with 10% FBS at 37 °C and 5% CO_2_.

For imaging experiments, cells were seeded at an appropriate density (~10,000–50,000 cells/mL) in 200 µL of complete medium in either 14 mm or 20 mm glass-bottom dishes (Cellvis, Mountain View, CA, USA). After 4 h, an additional 800 µL of medium was added, and cells were maintained overnight before acute treatment experiments or treated for 24–48 h with DMSO or the drugs listed in Table S1 (Supplemental Materials). Biological control dishes were included in all experiments.

**Figure 1:**
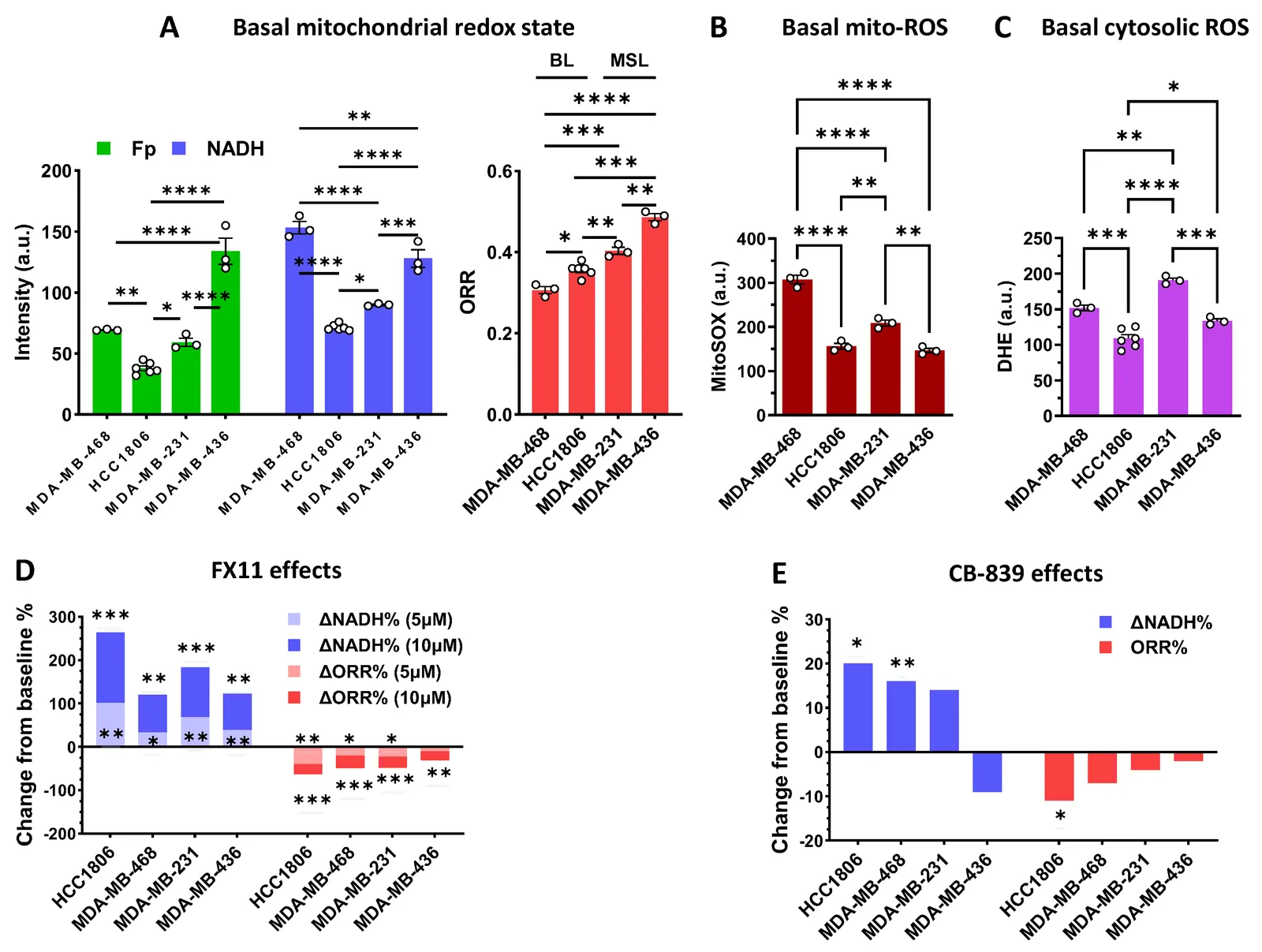
ORI reveals metabolic heterogeneity and differential responses to acute glycolytic and glutaminolytic perturbation in TNBC cells. (**A**–**C**) Baseline Fp, NADH, ORR, mitochondrial ROS (MitoSOX), and cytosolic ROS (DHE) in TNBC cells in LCIS^+^ (containing 0.1% DMSO). Each open circle represents an individual dish; (**D**) ORI responses to acute treatment (<5 min) with 5 or 10 µM FX11. (**E**) ORI responses to treatment with 1 µM CB-839 for 2 h. Percent change from baseline was calculated as (V_treated_ − V_baseline_)/V_baseline_ × 100%; n ≥ 3 dishes for each treatment in (**D**,**E**). *, *p* < 0.05; **, *p* < 0.01; ***, *p* < 0.001; ****, *p* < 0.0001.

The inhibitor concentrations were selected based on preliminary experiments and published studies. FX11 was evaluated at 5 and 10 µM for acute ORI responses (Figure 1D), and 5 µM was selected for subsequent experiments as the lower concentration that produced a pronounced metabolic response without the overt adverse effects on cell morphology observed at higher concentrations in preliminary experiments. For CB-839, preliminary acute experiments showed comparable ORI responses at 1 and 2 µM; therefore, 1 µM was selected for subsequent experiments, consistent with its established activity in TNBC models [36]. These concentrations were selected to produce measurable pathway-specific metabolic perturbations rather than to establish optimal therapeutic doses.

For combination treatment experiments, Taxol was used at cell line-specific concentrations selected based on preliminary optimization and published studies. HCC1806 cells were treated with 1 nM Taxol [28], whereas MDA-MB-231 cells were treated with 10 nM Taxol [37,38,39].

### 2.2. Imaging and Data Processing

ORI acquisition and data processing have been described in detail in our previous works [27,28,40,41] and in the Supplemental Materials. Imaging experiments were performed using two fluorescence microscope platforms due to instrument availability. For the datasets shown in Figure 1, images were acquired using a DeltaVision deconvolution microscope (Applied Precision, Issaquah, MA, USA). All remaining imaging experiments were performed using a Zeiss Observer 7 widefield fluorescence microscope (Zeiss, White Plains, NY, USA). Within each experiment, all treatment groups and controls were imaged using the same instrument and acquisition settings listed in Table S2. Because ORI metrics were analyzed and compared within individual experiments, the use of two imaging platforms did not affect the interpretation of treatment-induced changes.

Approximately 30 min prior to imaging, the complete medium was aspirated, and cells were rinsed twice with phosphate-buffered saline (PBS; pH 7.4) and then maintained in 1 mL of Live Cell Imaging Solution (Invitrogen™, ThermoFisher Scientific, Carlsbad, CA, USA; Cat# A59688DJ) supplemented with 11 mM glucose and 2 mM L-glutamine (LCIS^+^) during the imaging session.

For prolonged-treatment experiments, the medium was aspirated, and cells were washed twice with PBS and then imaged in LCIS+ without drug presence. In acute-treatment experiments (~3 h), the indicated agents were added directly to the dishes during the imaging session.

Following ORI, the same dishes were subsequently stained with either MitoSOX Red, dihydroethidium (DHE), or tetramethylrhodamine ethyl ester (TMRE) to assess mitochondrial superoxide, cytosolic ROS, or mitochondrial membrane potential (MMP), respectively. For proliferation assays, the same dishes that underwent ORI were subsequently stained with Hoechst 33342, followed by tile imaging of the entire dish and cell quantification using FIJI (ImageJ, 1.54r).

The raw images were processed using a customized MATLAB^®^ (Version 2019a, The MathWorks, Inc., Natick, MA, USA) program with background subtraction and thresholding at a signal-to-noise ratio (SNR) of 7.5 for the ORI metrics and an SNR of 5 for imaging of the fluorescent tracers [28,41]. For dish-based analyses, mean signal values were calculated for each FOV and averaged across FOVs (n ≥ 3 FOVs) within each dish to obtain dish means, which were then used for group-level comparisons (n ≥ 3 dishes). For selected experiments assessing pronounced acute cellular responses, FOV-level measurements were used to characterize within-culture response distributions, as indicated in the corresponding figure legends. Thus, each data replicate reflects a large, well-sampled cell population, which directly captures the substantial cell-to-cell metabolic heterogeneity, reduces measurement noise, and yields stable estimates. Data were normalized to the mean of the corresponding control group and expressed as fold change.

### 2.3. Seahorse Assays

HCC1806 cells were treated with 5 µM FX11 or 1 µM CB-839 for 24 h in 6-well plates. Cells were then washed with PBS and transferred to Seahorse XFe96 microplates at 15,000 cells per well (4 replicate wells per condition) in drug-free assay medium. The plate was centrifuged at 200× *g* for 2 min without brake to facilitate attachment. Mito Stress and Glycolysis Stress assays were performed according to standard Seahorse protocols using an Agilent XFe96 Analyzer (Agilent Technologies, Santa Clara, CA, USA). For the Mito Stress Test, oligomycin (1.5 µM), BAM15 (3 µM), and rotenone/antimycin A (1 µM) were sequentially injected. For the Glycolysis Stress Test, glucose (10 mM), oligomycin (1.5 µM), and 2-deoxy-D-glucose (2-DG; 50 mM) were sequentially injected.

### 2.4. Statistical Analysis

Group means were compared using Student’s t-test or one-way ANOVA, as appropriate. Post hoc multiple comparisons were performed using Tukey’s test for pairwise comparisons, Dunnett’s test for comparisons to a control group, or Šidák’s test, depending on the experimental design. Statistical analyses and graphing were performed using GraphPad Prism (v10; GraphPad Software, San Diego, CA, USA). Data are presented as mean ± SD for dish-based analysis or mean ± SEM for field of view (FOV)-based analysis. A *p* value < 0.05 was considered statistically significant.

## 3. Results

### 3.1. Basal Metabolic States and Responses to Metabolic Perturbation Across TNBC Cell Lines

We first examined the differences in the basal metabolic state of the four selected TNBC cell lines. Representative raw and processed images are shown in the Supplementary Materials (Figure S1). Characterization of ORI responses to mitochondrial uncoupler and electron transport chain inhibitors in these TNBC cell lines has been reported previously [42]. Figure 1 summarizes the quantified redox indices. Figure 1A shows that the two basal-like (BL) cell lines, MDA-MB-468 (BL1) and HCC1806 (BL2), exhibited a more reduced metabolic state, as indicated by lower ORR values, compared with the mesenchymal stem-like (MSL) cell lines MDA-MB-231 and MDA-MB-436, which exhibited a more oxidized metabolic state. Figure 1B,C display the basal mitochondrial and cytosolic ROS levels in these cell lines. Notably, the ROS profiles did not mirror the ORI-derived redox patterns, indicating that basal ROS abundance and redox state are not necessarily coupled across TNBC subtypes. These data show that ORI is sensitive to the basal metabolic differences in TNBC cells cultured in LCIS^+^, which contains only glucose (11 mM) and L-glutamine (2 mM) as the primary nutrients fueling glycolysis and glutaminolysis, respectively.

We next examined how the TNBC panel responded to glycolytic or glutaminolytic perturbation. FX11 is an inhibitor of lactate dehydrogenase A (LDHA), which catalyzes the terminal conversion of pyruvate to lactate, coupled to the oxidation of NADH to NAD^+^, thereby sustaining glycolytic flux [43]. CB-839 is a selective inhibitor of glutaminase 1 (GLS1), which catalyzes the conversion of glutamine to glutamate, a key step in glutaminolysis that supports anaplerosis and redox homeostasis [36,44]. GLS1 controls the entry of glutamine-derived carbon into central metabolic pathways. We observed that acute treatment with FX11 (<5 min) or CB-839 (2 h) induced differential ORI responses among the four TNBC lines (Figure 1D,E). HCC1806 cells showed the greatest response to both glycolytic and glutaminolytic inhibition compared to the other cell lines. MDA-MB-436 cells, in contrast, showed the smallest responses to both inhibitors.

These findings reveal intrinsic metabolic heterogeneity and differential responses to metabolic inhibition in TNBC cells, highlighting the utility of ORI as a functional phenotyping tool and motivating further investigation into the mechanisms underlying these redox responses.

### 3.2. Acute ORI Dynamics and Associated Redox Changes in HCC1806 Cells

Since HCC1806 cells exhibited the strongest acute ORI responses to both FX11 and CB-839 among the TNBC panel, we selected this line for further cellular analysis. As both glucose and glutamine contribute to mitochondrial NADH and FADH_2_ production, ORI reports the integrated cellular metabolic state arising from multiple metabolic inputs. Addition of glucose or glutamine to nutrient-starved cells produced the expected changes in ORI metrics. Both nutrients increased NADH levels, with glucose eliciting a greater increase than glutamine (Figure S2).

**Figure 2:**
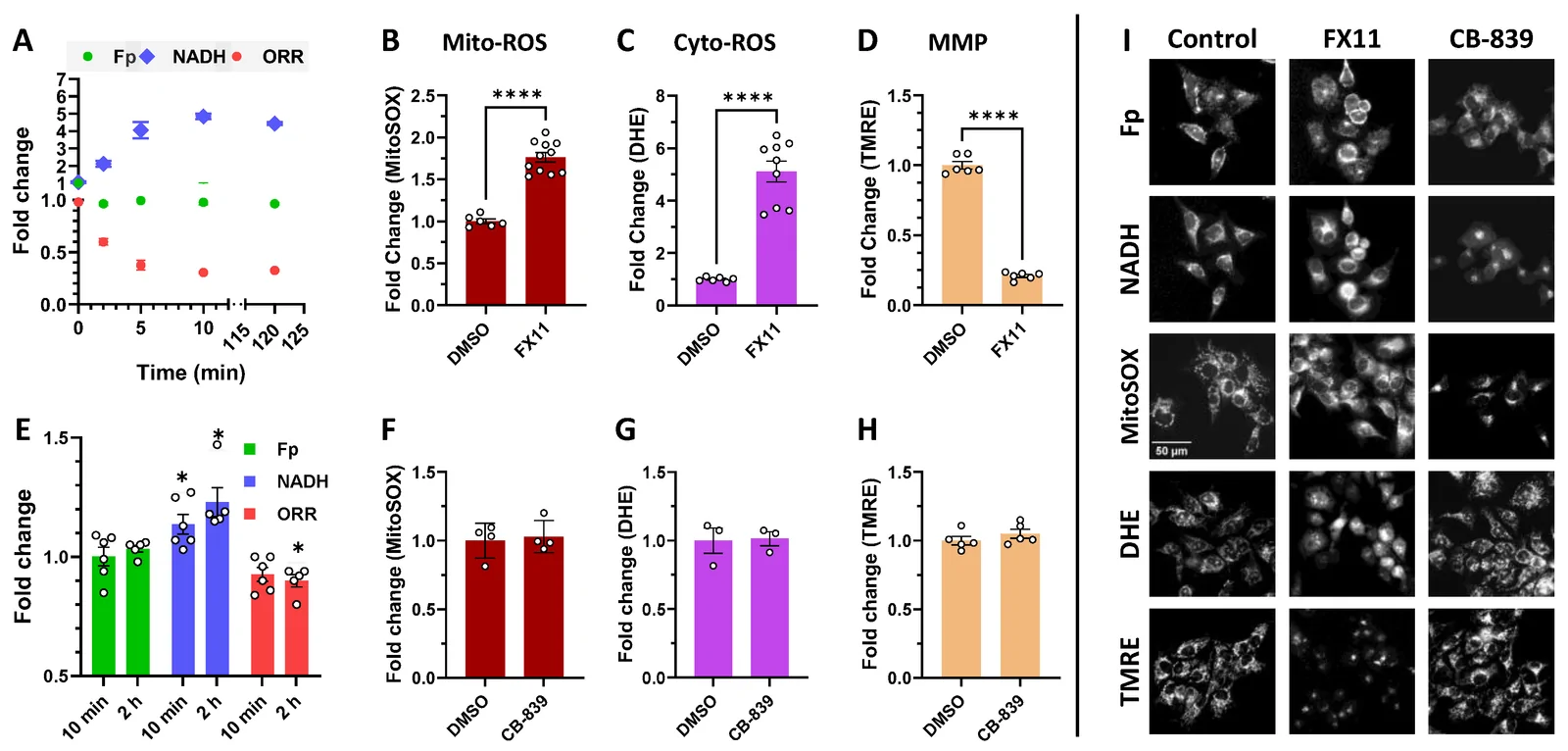
Acute effects of FX11 and CB-839 on HCC1806 cells. (**A**) Temporal in redox indices, following treatment with 5 µM FX11 (for each time point, n = 3~6 FOVs); (**B**–**D**) Acute FX11-induced changes in mito-ROS, cytosolic ROS, and MMP; (**E**) ORI response to acute treatment with 1 µM CB-839; (**F**–**H**) Mito-ROS, cytosolic ROS, and MMP following acute CB-839 treatment. (**I**) Representative fluorescence images of Fp, NADH, MitoSOX, DHE, and TMRE following control, FX11, or CB-839 treatment. Each open circle represents an FOV in (**B**–**D**) and an individual dish in (**E**–**H**). The responses shown in (**B**–**D**) were reproduced in an independent experiment. *, *p* < 0.05; ****, *p* < 0.0001.

We then assessed how acute FX11 or CB-839 treatment affected mitochondrial redox state and function by first collecting ORI signals and then staining and imaging the same dishes with the relevant fluorescent probes. Figure 2A shows the acute redox response from 2 min to 2 h following FX11 addition, demonstrating a marked increase in NADH signals and a corresponding decrease in ORR, while Fp signals remained stable during the first 10 min. Thereafter, the changes in the redox indices became more gradual.

To investigate the cellular correlates of this response, we subsequently quantified mitochondrial ROS, cytosolic ROS, and mitochondrial membrane potential (MMP). Acute FX11 treatment was associated with increased mitochondrial ROS (Figure 2B), an approximately five-fold increase in cytosolic ROS (Figure 2C), and a marked reduction in MMP (Figure 2D). The diffuse MitoSOX staining pattern (Figure 2I) observed following FX11 treatment is consistent with altered mitochondrial integrity and the reduction in MMP [45,46].

In contrast to FX11, acute CB-839 treatment induced more modest changes in NADH and ORR. CB-839 did not significantly alter mitochondrial ROS, cytosolic ROS, or MMP under these conditions. Consistent with these findings, MitoSOX and TMRE staining patterns remained largely unchanged following CB-839 treatment. Together, these results suggest that the acute ORI response to CB-839 occurs in the absence of the pronounced mitochondrial perturbation observed following FX11 treatment.

These data demonstrate that distinct acute ORI responses can arise from different underlying cellular processes. In HCC1806 cells, FX11 produced rapid changes in redox state that were accompanied by ROS accumulation and loss of MMP, whereas CB-839 elicited a more modest ORI response with minimal effects on these mitochondrial parameters.

### 3.3. Prolonged Metabolic Inhibition Induces Distinct Redox and Metabolic Responses

**Figure 3:**
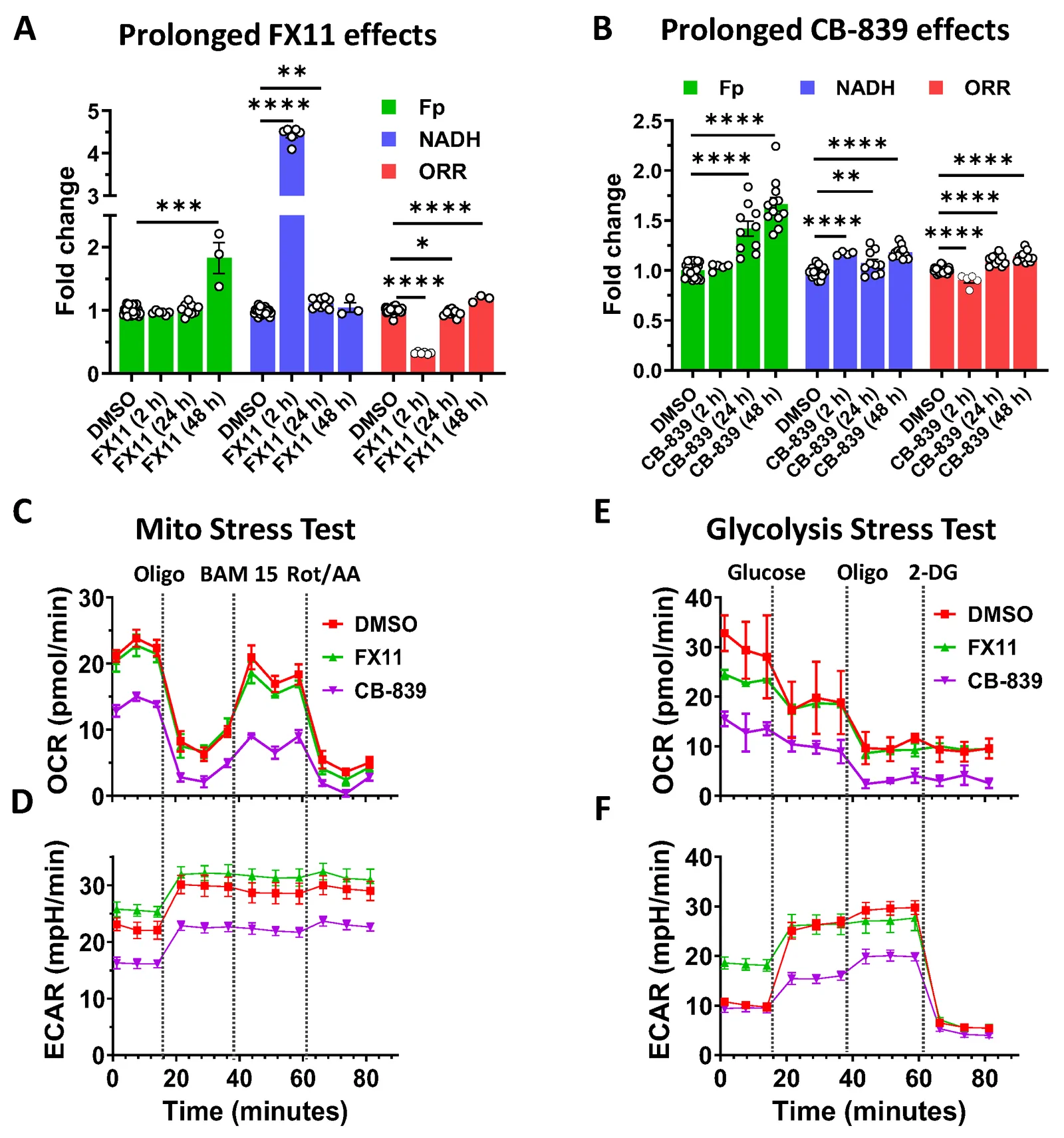
Prolonged metabolic responses to FX11 and CD-839 in HCC1806 cells. (**A**,**B**) ORI responses following 2, 24, and 48 h treatment with 5 µM FX11 (**A**) or 1 µM CB-839 (**B**); (**C**,**D**) OCR and ECAR measured by the Seahorse Mito Stress Test; (**E**,**F**) measured by the Seahorse Glycolysis Stress Test. Seahorse assays were performed follooing 24 h treatment with either 5 µM FX11 or 1 µM CB-839. Each open circle in (**A**,**B**) represents one individual dish and each solid dot in (**C**–**F**) represents the mean of four replicate wells in a 96-well Seahorse plate. *, *p* < 0.05; **, *p* < 0.01; ***, *p* < 0.001; ****, *p* < 0.0001.

We next examined prolonged treatment effects of FX11 and CB-839 on HCC1806 cells. Following FX11 treatment, NADH was only slightly elevated relative to the control at 24 h while Fp and ORR showed no significant change from control. By 48 h, Fp increased markedly (by ~75%) while NADH returned to baseline, resulting in an increase in ORR and indicating a shift from an initial reductive state to a more oxidized state (Figure 3A). These findings demonstrate that ORI can capture rapid, time-resolved metabolic adaptations and reveal distinct temporal phases of redox response. This temporal shift from an initially reduced to a more oxidized state suggests that the mitochondrial redox response to FX11 evolves during prolonged treatment.

Unlike FX11, which produced a time-dependent biphasic redox response characterized by an early reductive shift followed by a more oxidized state at 48 h, CB-839 induced a sustained increase in Fp and ORR throughout the prolonged treatment period (Figure 3B).

To further characterize the cellular state associated with prolonged metabolic inhibition, mitochondrial ROS, cytosolic ROS, and MMP were assessed following 24 h treatment. Unlike the pronounced acute increase in cytosolic ROS observed following FX11 treatment, cytosolic ROS returned to control levels by 24 h. FX11 produced only a modest increase in mitochondrial ROS and reduced MMP, whereas CB-839 had minimal effects on mitochondrial and cytosolic ROS but increased MMP (Figure S3).

To characterize metabolic flux associated with the prolonged-treatment response at 24 h, we measured OCR and ECAR using Seahorse Mito Stress and Glycolysis Stress assays following 24 h treatment with FX11 or CB-839. The Mito Stress assay showed that 5 µM FX11 treatment for 24 h did not affect OCR in HCC1806 cells but resulted in slightly higher ECAR throughout the assay (Figure 3C,D). Furthermore, HCC1806 cells exhibited a minimal spare respiratory capacity (SRC), defined as the difference between basal OCR and maximal OCR following BAM15 addition. Minimal SRC suggests limited mitochondrial respiratory reserve and adaptive capacity.

The Glycolysis Stress Test includes a pre-glucose phase in which the medium contains no glucose but does contain 2 mM glutamine. During this phase, FX11-treated cells exhibited lower OCR and higher ECAR than control cells (Figure 3E,F), indicating altered metabolic activity even when glutamine was the primary exogenous carbon source. Following glucose addition, OCR decreased to a similar level in both control and FX11-treated cells, accompanied by a marked increase in ECAR, consistent with a Crabtree-like metabolic response. Subsequent addition of oligomycin A further reduced OCR in both groups, consistent with inhibition of mitochondrial ATP synthesis. ECAR remained largely unchanged following oligomycin A treatment, with only a modest increase observed in control cells, suggesting limited glycolytic reserve in HCC1806 cells.

In contrast to FX11, CB-839 treatment for 24 h reduced both OCR and ECAR throughout the Mito Stress Test (Figure 3C,D), consistent with reduced mitochondrial respiration and glycolytic activity. In the Glycolysis Stress Test, CB-839-treated cells exhibited markedly lower OCR during the pre-glucose, glutamine-only phase compared to control cells (Figure 3E), indicating a strong reliance of HCC1806 cells on glutamine-supported mitochondrial respiration. Meanwhile, ECAR increased substantially in control cells but to a much lesser extent in CB-839-treated cells following glucose addition (Figure 3F).

Notably, CB-839-treated cells exhibited ECAR values similar to controls during the pre-glucose phase. Because glucose was absent during this phase, these data suggest that glutaminolysis inhibition suppresses mitochondrial respiration without substantially affecting baseline extracellular acidification. These findings indicate that GLS1 inhibition attenuates the increase in ECAR induced by glucose addition and mitochondrial ATP synthase inhibition.

Together, these results show that prolonged CB-839 treatment reduces both OCR and ECAR while inducing a more oxidized mitochondrial redox state. ORI and Seahorse therefore provide complementary views of the metabolic consequences of prolonged glutaminolysis inhibition.

### 3.4. Functional Consequences of ORI-Defined Metabolic Perturbations

**Figure 4:**
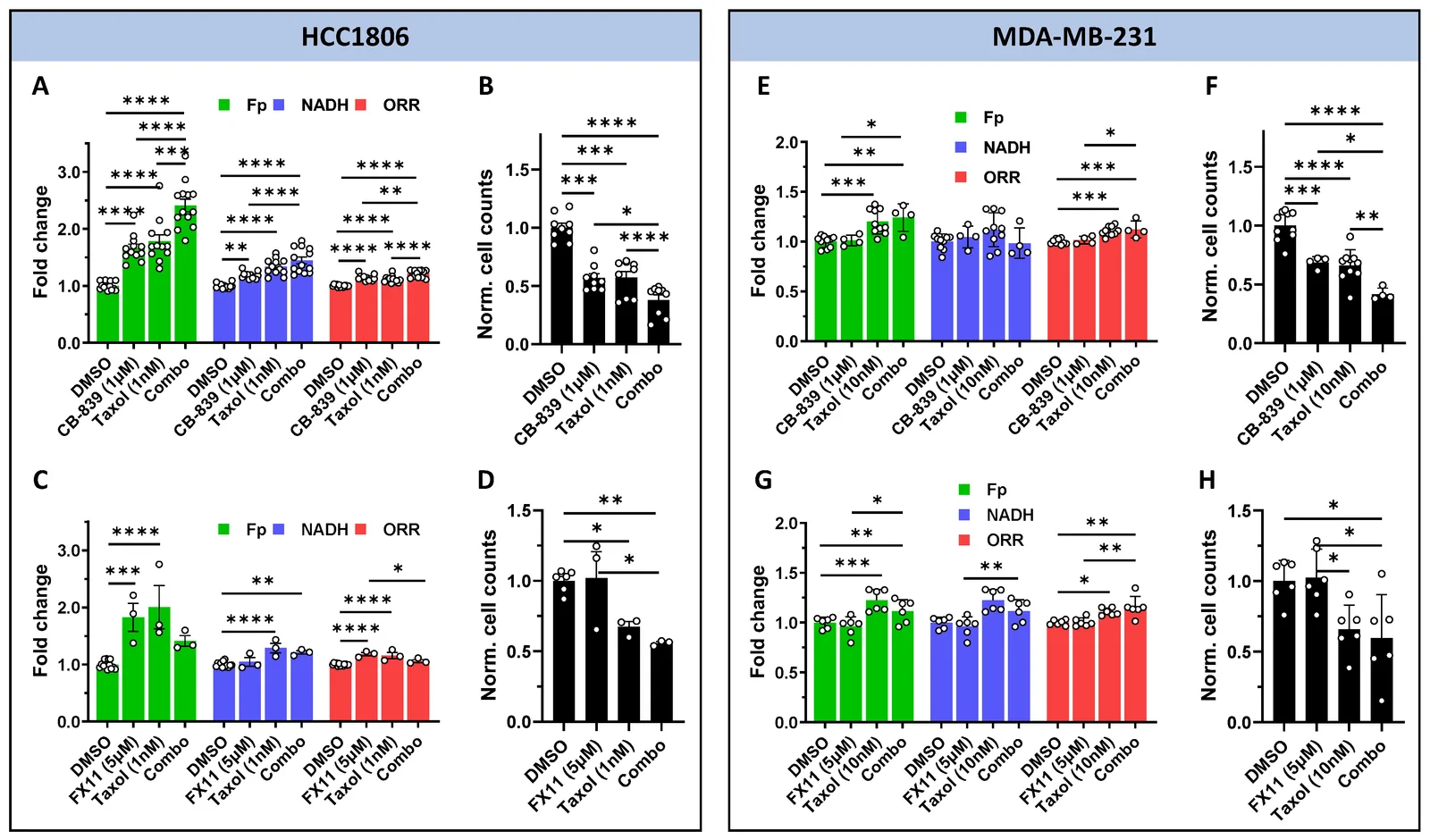
Differential ORI responses and proliferative effects of metabolic inhibition and combination treatment in HCC1806 and MDA-MB-231 cells. Cells were treated with FX11, CB-839, Taxol, or the indicated combinations for 48 h. ORI metrics (Fp, NADH, ORR) were first acquired, and cell numbers were subsequently quantified from the same dishes by cell quantification using Hoechst 33342-stained nuclei. (**A**–**D**) HCC1806 cells treated with CB-839 and/or Taxol (**A**,**B**) or FX11 and/or Taxol (**C**,**D**). (**E**–**H**) MDA-MB-231 cells treated with CB-839 and/or Taxol (**E**,**F**) or FX11 and/or Taxol (**G**,**H**). Data are presented as mean ± SD, with each open circle representing an individual dish. Statistical significance was assessed using one-way ANOVA followed by Tukey’s multiple comparisons test. *, *p* < 0.05; **, *p* < 0.01; ***, *p* < 0.001; ****, *p* < 0.0001.

Based on the distinct redox and metabolic responses induced by FX11 and CB-839, we next assessed whether these metabolic phenotypes translated into differential responses to metabolic inhibition and Taxol treatment in HCC1806 and MDA-MB-231 cells. Figure 4 summarizes the ORI responses and proliferative effects following 48 h treatment with CB-839, FX11, Taxol, or their combinations in HCC1806 and MDA-MB-231 cells. In HCC1806 cells, both CB-839 and Taxol induced significant redox changes, with increases in Fp, NADH, and ORR, and the combination treatment further enhanced these responses (Figure 4A). Following ORI measurements, the same dishes were stained with Hoechst 33342 and imaged for cell quantification. Both CB-839 and Taxol alone reduced cell numbers by approximately 50%, whereas the combination treatment produced significantly greater proliferative inhibition, reducing cell numbers by more than 60% (Figure 4B). Correlation analyses revealed significant associations between ORI metrics and cell number following CB-839-based treatments (Figures S4 and S5).

In contrast, FX11 increased Fp and induced a more oxidized redox state but did not significantly affect cell proliferation. Consistent with this observation, the FX11 + Taxol combination did not further enhance redox alterations or proliferative inhibition in HCC1806 cells (Figure 4C,D).

Compared with HCC1806 cells, MDA-MB-231 cells were less responsive to prolonged glycolytic or glutaminolytic perturbation. Neither CB-839 nor FX11 produced significant changes in ORI metrics in MDA-MB-231 cells (Figure 4E,G). CB-839 and Taxol each reduced cell numbers by approximately 30%, and the combination treatment resulted in significantly greater proliferative inhibition (Figure 4F). In contrast, FX11 did not significantly affect cell proliferation and did not enhance the response to Taxol (Figure 4H).

Together, these findings demonstrate that ORI-defined metabolic perturbations are associated with distinct proliferative responses following pathway-specific inhibition. Importantly, the magnitude of the ORI response did not necessarily correspond to the extent of proliferative inhibition, indicating that metabolic responsiveness and proliferative vulnerability represent related but distinct aspects of cellular metabolic behavior. These results support the utility of ORI as a metabolic phenotyping tool that links redox responses to proliferative outcomes following metabolic and chemotherapeutic perturbation.

## 4. Discussion

TNBC comprises metabolically heterogeneous subtypes with distinct pathway dependencies and therapeutic susceptibilities. In this study, we integrated optical redox imaging (ORI), Seahorse metabolic flux analysis, and imaging-based cell quantification to characterize metabolic heterogeneity and responses to glycolytic and glutaminolytic perturbation in TNBC cells. The study was designed sequentially, beginning with characterization of basal phenotypes and acute metabolic responses across four TNBC cell lines, followed by detailed cellular and metabolic characterization in HCC1806 and functional comparison of HCC1806 and MDA-MB-231 as representative, metabolically distinct models. Our results demonstrate substantial heterogeneity in basal redox phenotypes and acute metabolic responsiveness among TNBC models. Analyses in the representative models further revealed distinct temporal redox responses, associated changes in metabolic flux, and differential responses to metabolic inhibition and chemotherapy. Together, these findings highlight the value of integrating redox imaging with complementary functional assays to characterize multiple dimensions of metabolic behavior in TNBC cells.

### 4.1. ORI Reveals Metabolic Heterogeneity and Differential Responses to Acute Metabolic Perturbations in TNBC Cells

Metabolic heterogeneity is a hallmark of TNBC and contributes to differences in growth, metastatic potential, and therapeutic response. Using ORI, we observed that the two MSL cell lines, MDA-MB-231 and MDA-MB-436, exhibited a more oxidized metabolic state than the basal-like cell lines MDA-MB-468 (BL1) and HCC1806 (BL2) (Figure 1A and Figure S2). Interestingly, neither mitochondrial nor cytosolic ROS levels followed the same pattern (Figure 1B,C), indicating that basal redox state and ROS abundance are not necessarily coupled across TNBC subtypes. These findings demonstrate that ORI captures intrinsic metabolic heterogeneity that is not fully reflected by conventional ROS measurements.

Metabolic plasticity refers to the capacity of cancer cells to dynamically rewire metabolic pathways, enabling adaptation to environmental stress and supporting survival, growth, and therapy resistance [47]. We observed that acute FX11-induced mitochondrial redox changes were dose-dependent and more pronounced in HCC1806 and MDA-MB-231 cells, whereas MDA-MB-468 and MDA-MB-436 cells were less affected (Figure 1D). The magnitude of redox index changes varied across TNBC lines exposed to the same metabolic perturbations, highlighting intrinsic cell line-specific differences in redox responses. Following acute CB-839 treatment, the metabolic states of two basal-like lines, HCC1806 and MDA-MB-468, were more affected than those of the other two lines, consistent with a greater capacity of the more aggressive MSL cells to maintain redox homeostasis following metabolic perturbation.

HCC1806 cells were sensitive to both FX11 and CB-839 perturbations, suggesting lower tolerance to pathway-specific metabolic stress, while MDA-MB-231 and MDA-MB-436 cells exhibited greater metabolic plasticity, consistent with reports that MDA-MB-231 and MDA-MB-436 cells exhibit reduced sensitivity to chemotherapeutic agents such as Taxol [48,49]. Consistent with these findings, our prior work showed that this panel of TNBC cell lines exhibits differential responses to perturbation of the NAD^+^ salvage pathway, with HCC1806 cells displaying the greatest changes in mitochondrial redox state [29].

Since glycolysis, glutaminolysis, and NAD^+^ salvage pathways converge on mitochondrial NADH and FAD redox pools, differential ORI responses reflect variations in electron supply, utilization capacity, and redox buffering across TNBC subtypes. These findings position ORI as a functional redox phenotyping tool capable of distinguishing basal metabolic phenotypes and quantifying acute cellular responses to metabolic stress.

### 4.2. ORI Reveals Distinct Temporal Redox Responses to Glycolytic and Glutaminolytic Inhibition

Acute FX11 treatment induced an immediate and sharp increase in mitochondrial NADH and decrease in ORR (Figure 2A), accompanied by marked accumulation of mitochondrial and cytosolic ROS and reduced mitochondrial membrane potential (Figure 2B–D), indicating an acute redox and bioenergetic perturbation. These findings are consistent with a rapid reduction in the rate of NAD^+^ regeneration following LDHA inhibition, leading to altered cellular redox balance and accumulation of ROS. This early progressive response highlights the ability of ORI to capture real-time (minute-scale) redox coupling between cytosol and mitochondria. The subsequent slower phase may reflect cellular responses that partially stabilize mitochondrial redox state following the initial perturbation.

With prolonged FX11 treatment (24–48 h), the initial increase in NADH became less pronounced, while ORR returned to baseline and subsequently increased, driven by a marked rise in Fp (Figure 3A). Our LC-MS analysis (Supplementary Figure S6) showed that approximately 78% and 56% of the initial 5 µM concentration remained after 24 h and 48 h incubation in cell-free culture medium, respectively. While these measurements do not directly assess intracellular drug concentrations or target engagement, the observed 22% decrease in FX11 concentration over the first 24 h would be expected to produce only a modest reduction in the predicted extent of LDHA inhibition (from ~38% to ~33%, assuming Ki ≈ 8 µM and NADH competitive inhibition [50]). Although FX11 concentration reduction was greater at 48 h, substantial amounts of FX11 remained present throughout the experiment. Furthermore, a significant increase in Fp was observed at 48 h, indicating that metabolic alterations persisted despite the partial reduction in extracellular FX11 concentration. Therefore, loss of extracellular drug alone is unlikely to fully explain the return of NADH levels toward baseline, suggesting that additional cellular responses, including metabolic adaptation, may contribute to the observed phenotype. Importantly, acute treatment with 3 µM FX11, a concentration comparable to the extracellular concentration remaining after 48 h incubation, induced a pronounced reductive ORI response in both HCC1806 and MDA-MB-231 cells (Figure S7). These findings indicate that an extracellular FX11 concentration of approximately 3 µM remains sufficient to elicit a pronounced reductive redox response, further supporting the conclusion that reduced extracellular drug concentration alone is unlikely to account for the altered prolonged redox phenotype.

In contrast, CB-839 targets glutaminolysis upstream of the TCA cycle, and its effects emerge more gradually as mitochondrial metabolism adapts to reduced glutamine-derived substrate availability. At the early 2 h time point, the increase in mitochondrial NADH and decrease in ORR, without detectable ROS accumulation or MMP loss following GLS1 inhibition (Figure 2E–H), suggest that disruption of glutamine utilization initially perturbs mitochondrial redox balance without causing overt mitochondrial dysfunction.

Prolonged CB-839 treatment increased Fp and shifted the mitochondrial redox state toward a more oxidized state in contrast to the initial reductive shift observed after acute treatment. This may be associated with sustained alterations in TCA cycle activity and electron transport, leading to changes in redox balance. At later stages, the emergence of mitochondrial ROS (Figure S4) suggests progressive disruption of redox homeostasis during prolonged GLS1 inhibition. Because glutaminolysis contributes to both TCA cycle activity and cellular antioxidant metabolism, reduced glutamine utilization may contribute to the observed oxidized redox state. Together, these findings indicate that GLS1 inhibition initially induces a compensated reductive shift under preserved redox homeostasis, followed by a progressive loss of redox balance and the onset of ROS accumulation.

Interestingly, the magnitude of the acute redox response did not directly correspond to the extent of long-term metabolic flux changes or proliferative inhibition. Under the conditions examined, FX11 produced the most pronounced acute redox perturbation and diminished MMP (Figure 2A–D), whereas CB-839 produced greater effects on OCR and ECAR (Figure 3C–F) in HCC1806 cells and greater proliferative inhibition in the two models evaluated (Figure 4B,F). These findings suggest that acute metabolic responsiveness and long-term functional consequences represent distinct aspects of cellular metabolic behavior.

Although both ORI and Seahorse assays have been widely used to study cellular metabolism, there have been relatively few reports integrating these approaches [51,52]. Here, we show that ORI and Seahorse provided distinct but overlapping perspectives on metabolic regulation in HphoCC1806. ORI was particularly sensitive to rapid and time-dependent changes in cellular redox state, whereas Seahorse quantified population-averaged metabolic flux through mitochondrial respiration and glycolysis. The differing responses observed following FX11 and CB-839 treatment illustrate that substantial redox perturbation can occur in the absence of major changes in OCR or ECAR, and conversely, that suppression of metabolic flux may emerge following relatively modest acute redox responses. Together, these findings highlight the value of combining ORI and Seahorse measurements to capture complementary dimensions of metabolic behavior, including redox state, metabolic flux, and temporal responses to pathway-specific perturbation.

### 4.3. Functional Significance of ORI-Defined Metabolic Perturbations

We evaluated the functional significance of the ORI-defined metabolic responses by examining the effects of metabolic inhibition on cell number and Taxol response. Despite producing the largest acute redox perturbation, FX11 exerted minimal effects on cell number in both HCC1806 and MDA-MB-231 cells and did not substantially enhance the response to Taxol. In contrast, CB-839 significantly reduced cell number, particularly in HCC1806 cells, and further enhanced the effect of Taxol. These findings indicate that the magnitude of an acute redox response does not necessarily predict the degree of proliferative inhibition.

The differing responses to FX11 and CB-839 likely reflect the distinct metabolic dependencies of these TNBC models. HCC1806 cells exhibited pronounced acute and prolonged responses to glutaminolytic inhibition and greater redox remodeling following CB-839 treatment. In contrast, MDA-MB-231 cells displayed comparatively modest ORI changes in response to prolonged treatment despite exhibiting reductions in cell number following CB-839-based treatments. Together, these observations suggest that ORI is particularly useful for identifying pathway-specific metabolic perturbations and characterizing cellular responses to metabolic stress, while functional assays remain necessary to determine whether those perturbations translate into therapeutic vulnerability.

We further show that inhibition of glutaminolysis by CB-839 significantly reduced cell number in both lines and further enhanced the effect of Taxol in HCC1806 cells, accompanied by increases in Fp, NADH, ORR, and mitochondrial ROS. Consistent with this observation, our previous work demonstrated that inhibition of the NAD^+^ salvage pathway using FK866 similarly reduced growth and enhanced Taxol cytotoxicity in HCC1806 cells [28]. In both studies, sustained changes in ORI metrics were associated with reduced cell number, suggesting that sustained ORI changes may provide useful context for interpreting proliferative responses under specific metabolic perturbations.

In contrast, prolonged treatment with 5 µM FX11 did not significantly affect cell proliferation despite inducing pronounced acute redox perturbation (Figure 4D,H). Notably, FX11 reduced invasion in the highly invasive MDA-MB-231 cells (Figure S8), indicating that metabolic perturbation can influence cellular behavior even in the absence of substantial proliferative inhibition. Together, these findings suggest distinct roles for glycolytic and glutaminolytic pathways in regulating proliferation and invasive behavior and further emphasize that metabolic perturbations can produce divergent functional outcomes depending on the pathway targeted and the cellular context.

### 4.4. Advantages and Limitations of Widefield, Intensity-Based Live-Cell ORI

In this study, we showed that widefield, intensity-based live-cell ORI captures rapid, minute-scale metabolic changes at the single-cell level with compartmental resolution. By combining ORI with fluorescence imaging using relevant tracers, we further quantified compartment-specific ROS, mitochondrial membrane potential, and cell number in the same dishes that were subjected to ORI. Performing these measurements in the same dishes minimizes inter-sample variability and enables direct correlation of mitochondrial redox dynamics with functional cellular readouts, potentially even at the single-cell or same-field level with careful alignment of imaging coordinates.

Together, these features highlight the utility of ORI as a real-time metabolic phenotyping tool capable of resolving distinct temporal phases of metabolic response that are not accessible with conventional endpoint or flux-based assays. Widefield ORI is a fast and readily accessible method for monitoring metabolic changes in 2D cultures and has recently been extended to assess treatment response and drug screening in patient-derived cancer organoids, in part due to its higher effect size compared to confocal and two-photon fluorescence imaging [24,53]. Furthermore, ORI is scalable and compatible with high-throughput screening. Unlike Seahorse assays, which provide population-averaged measurements of metabolic flux, ORI enables rapid, single-cell assessment of redox state and temporal responses to metabolic perturbation. The two approaches therefore provide complementary information regarding metabolic behavior.

A technical limitation of ORI is that it does not distinguish between NADH and NADPH due to their overlapping fluorescence spectra, such that both contribute to the detected NAD(P)H signal. However, NADPH fluorescence is generally much weaker, and NADH is thought to dominate the signal. Consistent with this, a previous in vivo two-photon fluorescence study has shown that although both NADH and NADPH are derived from glucose metabolism, liver tumor cells exhibit metabolic reprogramming that favors NADH production for energy generation over NADPH synthesis for redox maintenance [54]. Nevertheless, direct measurements of NADPH and glutathione redox state would further refine the mechanistic understanding.

This study is limited to established in vitro cell-line models, which do not fully recapitulate the complexity of tumors, including interactions with the tumor microenvironment and spatial variation in nutrient and oxygen availability. Future studies using patient-derived organoids, primary cells, and in vivo models will therefore be important for validating these metabolic responses under more physiologically relevant conditions and evaluating treatment-associated toxicity. Furthermore, metabolic flux was assessed by Seahorse analysis only after 24 h treatment and therefore does not directly characterize the metabolic flux associated with the 48 h ORI phenotype; extending flux measurements to later time points would further clarify the relationship between temporal redox trajectories and metabolic flux. In addition, although the primary objective of this study was to characterize metabolic heterogeneity among TNBC cell lines and their responses to glycolytic and glutaminolytic perturbation, incorporation of non-tumoral mammary epithelial models in future studies will be necessary to determine whether the observed metabolic responses are cancer-selective before conclusions regarding a potential therapeutic window can be drawn. Clinical translation of metabolism-targeted therapies remains challenging because metabolic dependencies can vary substantially among tumors and can change through compensatory pathway activation under therapeutic pressure. In addition, many metabolic pathways targeted in cancer are also required by normal tissues and immune cells, potentially limiting the therapeutic window through systemic toxicity. These considerations emphasize the need to define context-specific metabolic responses and evaluate tumor selectivity in physiologically relevant models before translating metabolic dependencies into therapeutic strategies.

Despite these limitations, ORI may provide a useful platform for evaluating metabolic interventions in more complex experimental systems. Metabolic targeting strategies are increasingly being explored in combination with immunotherapy [55,56]. For example, inhibition of glutamine metabolism has been shown to enhance tumor-specific immunity by modulating suppressive myeloid cells in a mouse TNBC model [57]. The methodological framework presented here may be extended to evaluate metabolic interventions in combination with checkpoint blockade immunotherapy or CAR T cell therapy in TNBC models [58].

More broadly, these findings support the integration of ORI with complementary functional measurements to relate dynamic redox responses to metabolic and cellular outcomes. Such multimodal assessment may improve characterization of context-dependent metabolic responses in heterogeneous cancer populations.

## 5. Conclusions

Our study demonstrates that TNBC cell lines exhibit pronounced metabolic heterogeneity, with differential redox responses to glycolytic and glutaminolytic perturbation that depend on treatment duration and metabolic context. Using the representative models HCC1806 and MDA-MB-231, we show that glutaminolytic inhibition by CB-839 reduces cell number and enhances the anti-proliferative effect of Taxol, while glycolytic and glutaminolytic inhibition produce distinct temporal redox responses and functional outcomes. These findings highlight the context-dependent nature of metabolic responses and functional outcomes in TNBC.

Methodologically, we demonstrate that wide-field optical redox imaging (ORI) can be integrated with complementary fluorescence imaging, Seahorse metabolic flux analysis, and imaging-based cell quantification to characterize multiple dimensions of metabolic behavior. This multimodal framework captures basal metabolic phenotype, pathway-specific responsiveness, temporal redox trajectories, and treatment-associated functional outcomes, providing a more comprehensive view of metabolic heterogeneity than any single assay alone.

Together, our findings indicate that metabolic responses in TNBC are highly context- and time-dependent and support the use of wide-field ORI as a rapid, readily-accessible, single-cell approach for characterizing metabolic phenotypes and responses to metabolic perturbation. More broadly, this framework may facilitate identification of biologically relevant metabolic phenotypes and inform the rational design of metabolism-targeted therapeutic strategies.

## Data Availability

The relevant data will be provided upon reasonable request.

## Supplementary Materials

The following supporting information can be downloaded at: https://www.mdpi.com/article/10.3390/metabo16090613/s1, Figure S1: Representative processed redox images and corresponding raw inset images of HCC1806 cells treated with DMSO, FX11, or CB-839; Figure S2: ORI responses to various metabolic modulations in HCC1806 cells; Figure S3: Effects of 24 h prolonged FX11 or CB-839 treatment on mito-ROS, cytosolic ROS, and MMP in HCC1806 cells; Figure S4: CB-839-based treatment effects on mitochondrial ROS and its correlations with cell number in HCC1806 cells following 48 h treatment; Figure S5: Linear regression analyses between cell number and ORI metrics in MDA-MB-231 cells following 48 h treatment with CB-839, Taxol, or their combination; Figure S6: Chemical stability of FX11 and CB-839 in cell-free complete RPMI 1640 medium determined by LC-MS; Figure S7: Pronounced reductive shift in HCC1806 and MDA-MB-231 cells induced by acute 3 μM FX11 treatment; Figure S8: Prolonged FX11 treatment effects on the invasive potential of MDA-MB-231 cells. Table S1: Compounds used in this study. Table S2: The Zeiss microscope settings.
