# Supplementary material for: Live-Cell Optical Redox Imaging Reveals Metabolic Heterogeneity and Context-Dependent Responses to Metabolic Perturbation in TNBC Cells
Source: Metabolites. 2026 Aug 27;16(9):613. doi: 10.3390/metabo16090613 (PMC13609622; doi:10.3390/metabo16090613)
Supplement: Supplementary file 1 [file metabolites-16-00613-s001.zip › metabolites-4469425-supplementary.pdf]

Materials and Methods

Table S1. Compounds used in this study

Reagent	Type	Vendor/Cat. #	Final Concentration
Paclitaxel	Chemotherapeutic	Cell Signaling Technology/ 9807S	1-10 nM
CB-839	Metabolic inhibitor of glutaminase 1 (GLS1)	Cayman Chemical/11969-10	1 μM
FX11	Metabolic inhibitor of lactate dehydrogenase A (LDHA)	MedChemExpress (Monmouth Junction, NJ, USA/HY-16214	5 & 10 μM
FCCP	Mitochondrial uncoupler	MilliporeSigma (St. Louis, MO, USA)/C2920	0.5 μM
Rotenone	Complex I inhibitor	MilliporeSigma (St. Louis, MO, USA)/R8875	1 μM
Antimycin A	Complex III inhibitor	MilliporeSigma (St. Louis, MO, USA)/A8674	1.25 μg/mL
MitoSOX™ Red	Fluorescent probe for mitochondrial ROS	Thermo Fisher Scientific (Waltham, MA, USA)/ M36008	2 μM
DHE	Fluorescent probe for cytosolic ROS	MilliporeSigma (St. Louis, MO, USA)/ D7008	2 μM
TMRE	Fluorescent probe for mitochondrial membrane potential	Thermo Fisher Scientific (Waltham, MA, USA)/ T669	200 nM
Hoechst 33342	Fluorescent nuclear stain	Thomas Scientific (Swedesboro, NJ, USA)/15547	10 μM

Imaging settings

For datasets shown in Figure 1A, 1D, and 1E, images were acquired using a DeltaVision deconvolution microscope maintained at 37 °C, with a 40× objective (NA = 0.95), a 512 × 512 pixel field of view, 2 × 2 binning, and a spatial resolution of 0.32 × 0.32 μm^2. Excitation filters of 360/40 nm (NADH) and 470/40 nm (Fp), and emission filters of 455/50 nm (NADH) and 520/40 nm (Fp) were used, with an exposure time of 3 s for both channels. Photosensor readings were incorporated during image processing to correct for fluctuations in the Xe arc lamp output.

The remaining imaging experiments were performed using a Zeiss wide-field fluorescence microscope (Zeiss, Observer 7, White Plains, NY, USA) maintained at 37 °C. Images were acquired with a 20× objective (NA = 0.8), yielding a FOV of 562.6 × 356.3 μm (1920 × 1216 pixels; 0.293 × 0.293 μm^2 per pixel). For all experiments, 3–15 randomly selected fields of view (FOVs) were imaged. FOV selection and focus was performed using transmitted light to avoid photobleaching. The imaging settings are listed in Table S2.

Table S2. The Zeiss microscope settings

Channel	Excitation (nm)	Emission (nm)	LED Power	Exposure (s)	Gain	Incubation (min)
NADH	370–400	414–450	25%	2	1	N/A
Fp	450–488	500–530	40%	3	1	N/A
TMRE	540-570	580-610	40%	0.05	1	20
DHE	540-570	580-610	50%	0.05	1	30
MitoSOX Red	370–400	580–610	40%	0.2	1	10
Hoechst 33342	370–400	414–450	50%	0.15	1	30

Supplementary figures

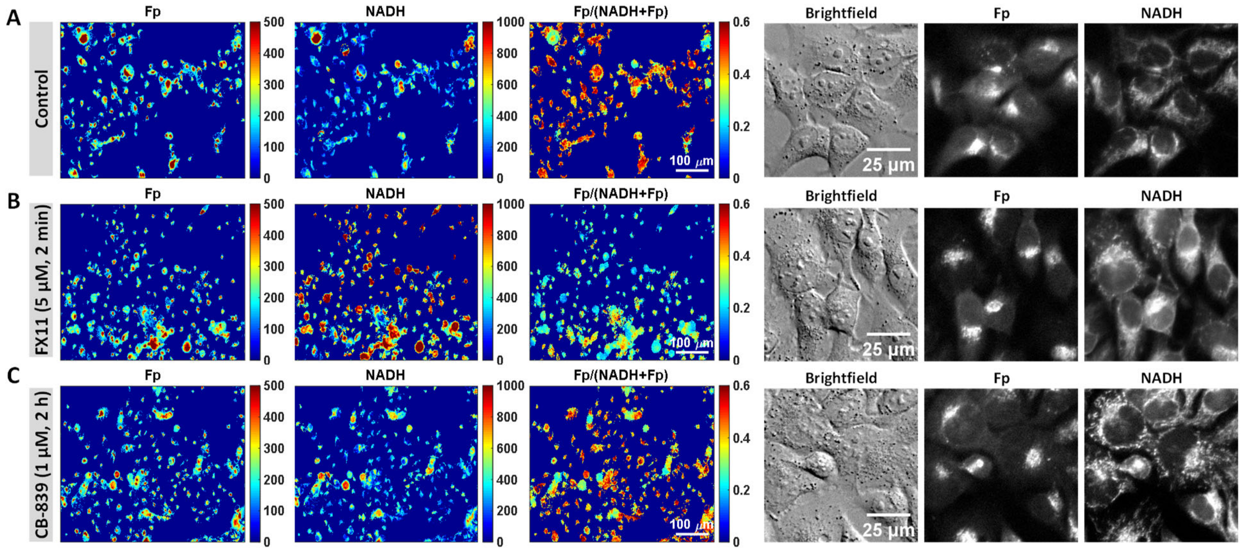

Figure S1. Representative processed redox images and corresponding raw inset images of HCC1806 cells treated with DMSO, FX11, or CB-839. A) Control cells treated with DMSO. B) Cells treated with 5 µM FX11 for 2 min. C) Cells treated with 1 µM CB-839 for ~2 h. Each panel includes processed images of Fp, NADH, and the optical redox ratio (ORR), along with corresponding raw inset images (brightfield, Fp, and NADH). Processed images include color bars indicating pixel intensity, with red representing higher intensity and blue representing lower intensity. The ORR (Fp/(NADH + Fp)) is normalized between 0 and 1 and displayed over a range of 0–0.6 for visualization. Higher ORR values indicate a more oxidized redox state, whereas lower values indicate a more reduced state.

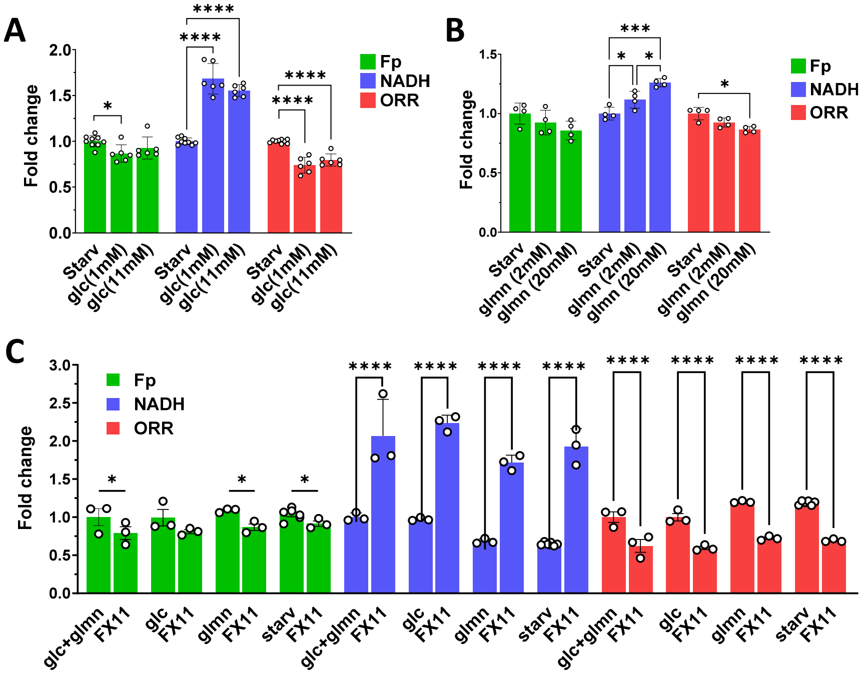

Figure S2. ORI responses to various metabolic modulations in HCC1806 cells. A) Redox restoration after spiking 1 mM glucose (glc) to cells maintained in PBS-only medium (Starv=nutrient starvation) for ~1 h; B) Cells in PBS-only medium were stimulated with 2 or 20 mM L-glutamine; C) Acute 5 µM FX11 treatment (<5 min) effects on cells maintained in various media. glc=glucose-only, glmn=glutamine-only, starv=PBS-only. Each open circle represents an individual dish. Statistical significance was assessed using one-way ANOVA followed by Tukey's multiple comparisons test. *, p<0.05; ***, p <0.001; ****, p <0.0001.

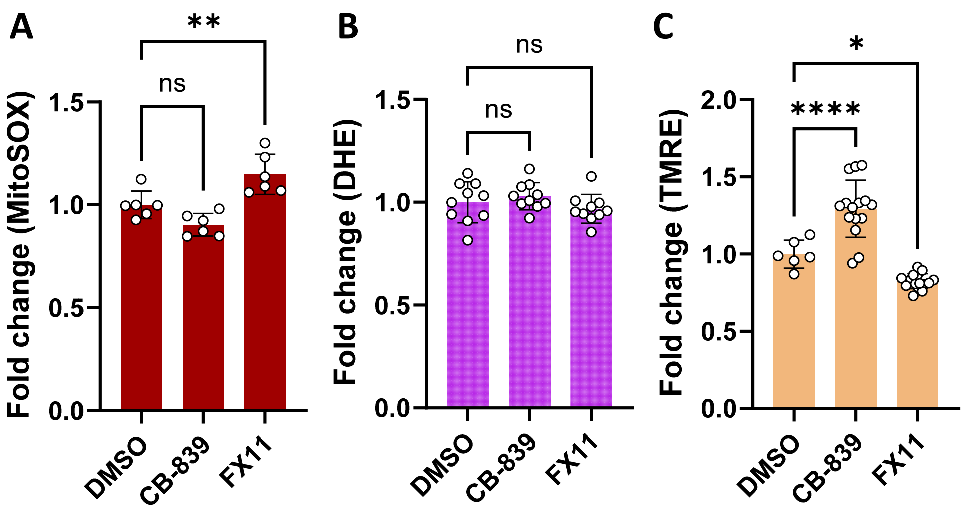

Figure S3. Effects of 24 h prolonged FX11 (5 µM) or CB-839 (1 µM) treatment on mitochondrial ROS (A), cytosolic ROS (B), and mitochondrial membrane potential (C) in HCC1806 cells. An open circle represents a FOV. Statistical significance was assessed using one-way ANOVA followed by Dunnett's multiple comparisons test. *, p <0.05; **, p <0.01; ****, p <0.0001.

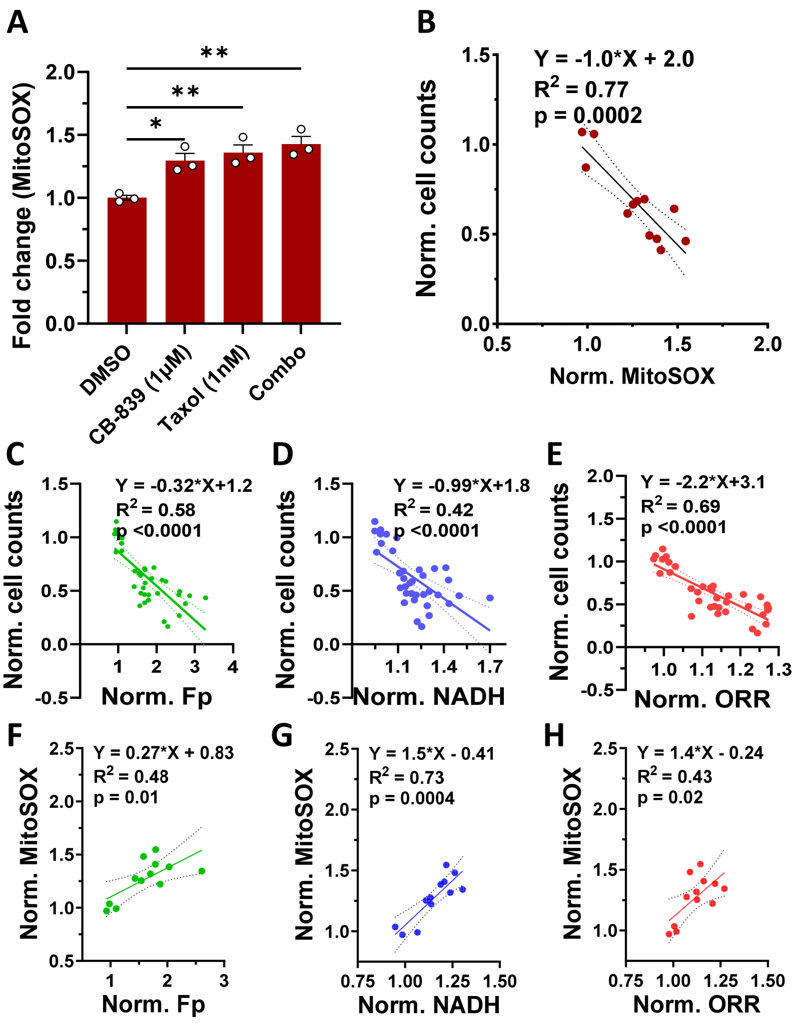

Figure S4. CB-839-based treatment effects on mitochondrial ROS and its correlations with cell number in HCC1806 cells following 48 h treatment. A) Changes in mitochondrial ROS due to various treatment. Statistical significance was assessed using one-way ANOVA followed by Dunnett's multiple comparisons test; B-E) Significant negative correlations between cell growth and mitochondrial ROS, Fp, NADH, ORR, respectively; F-H) Significant positive linear correlations between mitochondrial ROS and Fp, NADH, the ORR, respectively. Each open circle/dot represents a dish. All parameters are normalized relative to their respective controls. Linear equation, correlation coefficients (R^2) and corresponding *p* values are shown in the subfigures C-H. An open circle or dot represents a dish. *, p <0.05; **, p <0.01.

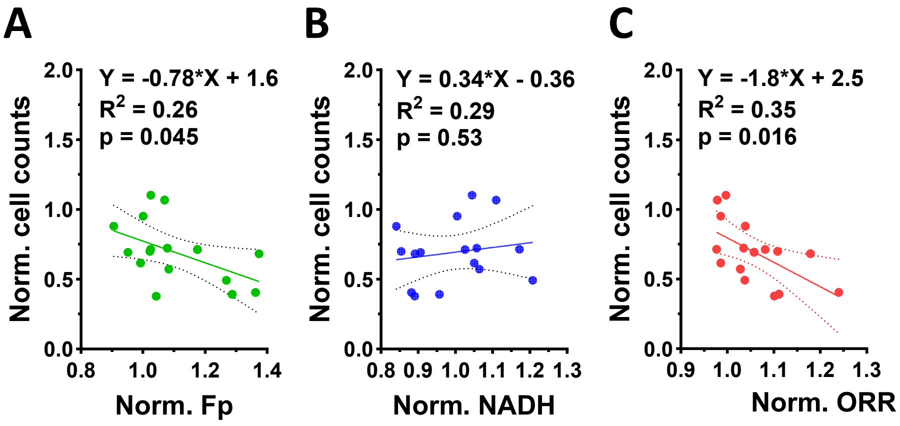

Figure S5. Linear regression analyses between cell number and ORI metrics in MDA-MB-231 cells following 48 h treatment with CB-839 (1 µM), Taxol (10 nM), or their combination. A) Fp negatively correlates with cell number; B) No significant correlation between NADH and cell number: C) ORR negatively correlates with cell number. Linear equation, correlation coefficients (R^2) and corresponding *p* values are shown in each subfigure.

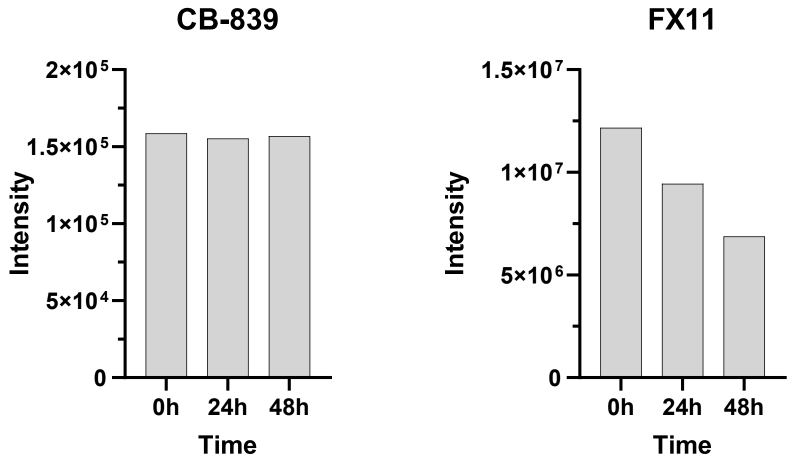

Figure S6. Chemical stability of FX11 and CB-839 in cell-free complete RPMI 1640 medium determined by LC-MS. CB-839 (1 µM) remained chemically stable for at least 48 h under the same incubation conditions used for the TNBC experiments. FX11 (5 µM) retained approximately 78% (corresponding to 3.9 µM) and 56% (corresponding to 2.8 µM) of its initial concentration after 24 h and 48 h incubation at 37 °C and 5% CO_2, respectively. LC-MS analysis was performed using a high-resolution Agilent 6546 Q-TOF LC-MS system coupled to an Agilent 1290 Infinity II UHPLC operating in negative ion mode with hydrophilic interaction liquid chromatography (HILIC).

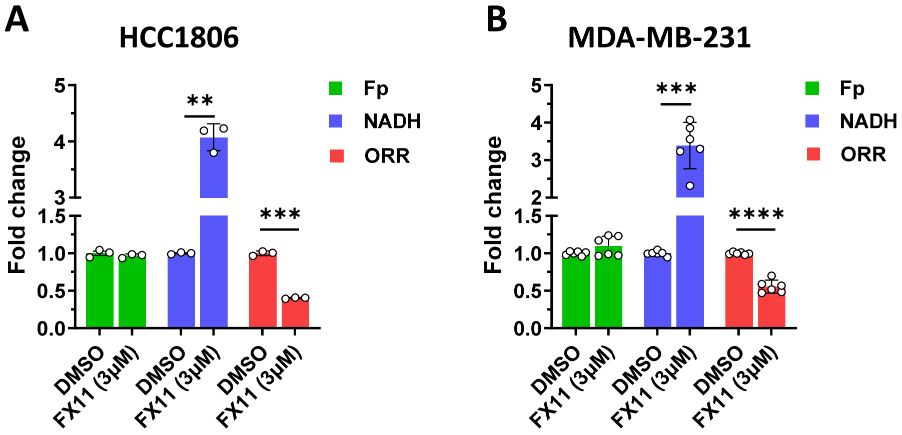

Figure S7. Pronounced reductive shift in HCC1806 and MDA-MB-231 cells induced by acute 3 µM FX11 treatment. A) HCC1806; B) MDA-MB-231. An open circle represents a dish. **, *p* <0.01, ***, *p* <0.001, ****, *p* <0.0001.

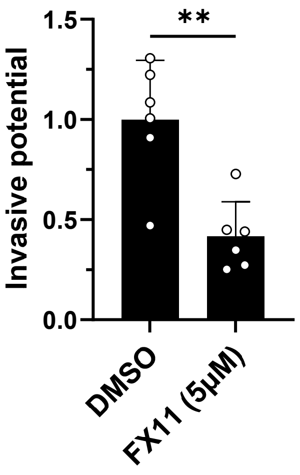

Figure S8. Prolonged FX11 treatment effects on the invasive potential of MDA-MB-231 cells. FX11 treatment for 48 h reduced the invasive potential of the highly metastatic MDA-MB-231 cells. Cell invasion was assessed using Matrigel (MilliporeSigma, Cat.#. CB356238) coated Transwell plate. Each dot represents a well. **, *p* <0.01.
